# Green Synthesis and Characterization of Konjac Glucomannan-Capped Cerium Nanoparticles for Photocatalytic Degradation of Naphthol Blue Black and Methyl Orange Dyes in Wastewater

**DOI:** 10.3390/nano16120739

**Published:** 2026-06-13

**Authors:** Juan José Andrade Sepúlveda, Javiera Moraga Muñoz, Pandian Lakshmanan, Kishor Kumar Sadasivuni, Saravanan Chandrasekaran, Diana Abril, Radha Devi Pyarasani, John Amalraj

**Affiliations:** 1 Facultad de Ciencias Agrarias y Forestales, Universidad Católica del Maule, Talca 3460000, Chile; juan.andrade@alu.ucm.cl (J.J.A.S.);; 2 Laboratory of Materials Science, Instituto de Química de Recursos Naturales, Universidad de Talca, 747, Talca 3460000, Chile; 3 Department of Chemistry, Tagore Engineering College, Rathinamangalam, Chennai 600127, India; plakshmanan@tagore-engg.ac.in; 4 Center for Advanced Materials, Qatar University, Doha P.O. Box 2713, Qatar; kishorkumars@qu.edu.qa; 5 Department of Chemistry, School of Engineering, Presidency University, Yelahanka, Rajanukunte, Bengaluru 560119, India; saravanan@presidencyuniversity.in; 6 Departamento de Biología y Química, Facultad de Ciencias Básicas, Universidad Católica del Maule, Talca 3466000, Chile; dabril@ucm.cl; 7 Centro de Investigación de Estudios Avanzados del Maule, Vicerrectoría de Investigación y Postgrado, Universidad Católica del Maule, Talca 3466000, Chile; 8 Laboratory of Nanomaterials, Centro de Investigación de Estudios Avanzados del Maule (CIEAM), Universidad Católica del Maule, Talca 3466000, Chile

**Keywords:** cerium nanoparticles, konjac glucomannan, adsorption, photocatalytic degradation, azo dyes, naphthol blue black, methyl orange

## Abstract

Green synthesis of KGM-capped CeO_2_ nanoparticles was successfully achieved through a simple coprecipitation method using Konjac Glucomannan (KGM) as a biopolymeric capping and stabilizing agent. The reaction conditions were optimized by varying pH (9–11) and temperature (30–70 °C) to evaluate their influence on nanoparticle formation and photocatalytic performance. The synthesized KGM–CeO_2_ nanoparticles were comprehensively characterized using FTIR, UV–Vis spectroscopy, XRD, SEM–EDS, TEM, DLS, and ZP analysis to investigate their structural, optical, morphological, and surface properties. The characterization results confirmed the successful formation of porous sponge-like branched CeO_2_ nanostructures with irregular morphology. XRD analysis revealed the crystalline nature of the nanoparticles with an average crystallite size of approximately 7.7 nm, while DLS analysis showed an average hydrodynamic particle size of 29.7 nm with a biomodal particle size distribution. The positive zeta potential value (+16.75 mV) confirmed good colloidal stability and reduced agglomeration due to effective capping by KGM. The synthesized nanoparticles also exhibited favorable optical properties with band gap values suitable for photocatalytic applications. The adsorption and photocatalytic degradation performance of the KGM–CeO_2_ nanoparticles was investigated against synthetic textile dyes, including Naphthol Blue Black (NBB), Methyl Orange (MO), and a mixed NBB–MO dye system under acidic conditions. Using an adsorbent dosage of 50 mg and dye concentrations of 100 mg/L, the material achieved degradation efficiencies of approximately 99% for NBB, 91% for MO, and 52% for the mixed dye system under UV irradiation for 120 min. Adsorption kinetic studies indicated that the pseudo-second-order model provided the best fit, suggesting that chemisorption is the dominant adsorption mechanism involving multifunctional surface interactions. These findings are particularly relevant for industrial wastewater treatment, since actual textile effluents typically contain complex mixtures of dyes and organic contaminants rather than single dye pollutants. The mixed dye experiments, therefore, provide a more realistic simulation of industrial wastewater conditions. Overall, the synthesized KGM–CeO_2_ nanoparticles demonstrate excellent potential as an eco-friendly, cost-effective, and sustainable multifunctional material for adsorption-assisted photocatalytic treatment of dye-contaminated wastewater. Further optimization of operational conditions and catalyst surface properties may enhance its efficiency in multicomponent wastewater systems.

## 1. Introduction

The rapid expansion of the textile, printing, and cosmetic industries has led to the significant discharge of hazardous organic pollutants into aquatic ecosystems. Among these, synthetic Azo dyes like Congo Red (CR), Naphthol Blue Black (NBB), and Methyl Orange (MO) are particularly concerning due to their complex aromatic structures, high toxicity, and resistance to natural degradation. The increasing presence of hazardous organic pollutants, particularly synthetic dyes, in industrial wastewater demands the development of efficient and sustainable treatment solutions [1,2,3]. Traditional wastewater treatment methods often fall short of complete mineralization, necessitating the development of advanced oxidation processes. Among various advanced oxidation processes, photocatalysis utilizing metal oxide nanoparticles has emerged as a promising approach due to their strong light absorption, stability, and redox properties [4,5]. Furthermore, it offers a sustainable pathway to degrade recalcitrant organic molecules into non-toxic substances like CO_2_ and H_2_O [3].

Among semiconductor photocatalysts, cerium oxide (CeO_2_) nanoparticles (NPs) have attracted considerable attention because of their unique physicochemical and redox properties. CeO_2_ is a rare-earth metal oxide capable of reversible switching between the trivalent (Ce^3+^, cerous) and tetravalent (Ce^4+^, ceric) oxidation states under oxidizing and reducing conditions [6,7]. At the nanoscale, CeO_2_ has a mixed state where both Ce^3+^ and Ce^4+^ can coexist on its surface. This unique redox property of ceria to store and release oxygen in its intrinsic structure makes it highly promising for catalytic applications [8,9]. The strong photocatalytic activity of CeO_2_ NPs is mainly attributed to their ability to generate reactive oxygen species (ROS) under irradiation, which effectively decompose recalcitrant organic pollutants [2]. Furthermore, the unique electronic structure of cerium facilitates oxygen vacancy formation, which significantly enhances its photocatalytic performance [10]. CeO_2_ NPs are characterized by a band gap energy of approximately 3.1 eV, as well as considerable thermal and chemical stability and relatively low cost [11,12]. Due to these properties, CeO_2_ has been explored for various applications such as electrical, electronic, catalytic, adsorption, optical, electrochemical, batteries, functional materials, energy storage, magnetic data storage, and sensing properties [13,14,15,16,17,18]. In particular, CeO_2_-NPs have shown promising results in the degradation of dyes such as methylene blue (MB) and methyl orange (MO) with around 90% degradation after 60 min under UV radiation [19,20,21]. Despite these advantages, widespread industrial application of the conventional synthesis of CeO_2_ NPs faces significant drawbacks. Traditional methods ranging from classical precipitation to hydrothermal, sol–gel, ball milling and microwave-assisted routes often rely on harmful organic solvents, toxic reducing agents, and hazardous organic templates, which can accumulate in the environment, causing implications for the health of living organisms and the environment [22,23,24,25,26,27,28]. Furthermore, pristine CeO_2_ possesses high surface energy and has technical challenges such as particle aggregation, which later diminishes its active surface area and limits its recyclability, often hindering its widespread industrial application.

To overcome these hurdles of environmental toxicity and particle clumping, current research focuses on green, bio-directed surface functionalization [6,19]. Green synthesis of CeO_2_ NPs using biological products, microbial biomass and plant extracts has been carried out and natural polysaccharides have emerged as sustainable alternatives [29,30]. Various biopolymers, including Agarose, fructose, starch, xanthan Gum, Dextran, Polyethylene glycol, Chitosan, and their derivatives, have also been used as stabilizers for the synthesis of CeO_2_ NPs [6,31,32,33,34,35]. Biopolymers possess abundant functional groups like –COOH, –OH, and –NH_2_, which can stabilize and cap metal ions, significantly improving particle size by preventing agglomeration and enhancing their physical-chemical properties [36,37,38,39,40]. The main role of a capping agent is to bind to metal ions and prevent nanoparticle agglomeration by mitigating their tendency to self-associate due to high surface energy. Capping agents also control the morphology of the nanostructure through their soft template effect and influence chemical kinetics. Each capping agent plays a different role and has distinct properties that affect their performance. Furthermore, the molecular weight of the capping agent significantly impacts the assembly behaviors of the nanoparticles, and this will depend on the properties of the capping agent, which are influenced by van der Waals interaction, capillary interaction, surface tension, hydrophobic interaction, and Hydrogen bonding [35]. Selecting the appropriate biopolymer matrix is essential for engineering ultrasmall ceria nanoparticles, but achieving comparable sub-10 nm dimensions through entirely green frameworks remains a challenge. Currently, the average particle size of CeO_2_ NPs synthesized using biopolymers as stabilizers is ~10 ± 5 nm in size. However, there is still potential to further reduce the particle size of CeO_2_ NPs by exploring new biopolymers as capping agents to achieve desired properties.

Among natural biopolymers, Konjac Glucomannan (KGM) has attracted increasing attention as an eco-friendly material. KGM is a natural polysaccharide comprising D-mannose and D-glucose units linked by β-(1, 4) bonds [41,42]. Due to the presence of abundant hydroxyl and carbonyl groups in their structure, KGM can easily take part in numerous chemical reactions, imparting superior properties compared to traditional materials. These functional groups enable KGM to form hydrogen bonds in aqueous solutions and have strong interactions with other macromolecules, thereby allowing it to absorb and retain large amounts of water and maintain gel stability. In addition, KGM has gained much attention due to its low cost, high viscosity, excellent film-forming ability, biocompatibility, biodegradability, renewability, and nontoxicity. It has been widely used in various applications, including food, pharmaceutical, antimicrobial, and chemical engineering applications [41,43]. Recently, KGM has also been employed as a reducing agent in the synthesis of Ag NPs [44,45] and Au NPs [46], demonstrating its effectiveness as a capping agent in metal nanoparticle synthesis. Therefore, the present study focuses on the green synthesis of CeO_2_ using KGM as a capping agent, aiming to leverage its stabilizing properties to mitigate aggregation and enhance photocatalytic degradation efficiency against challenging textile dyes like NBB and MO [4,19,47]. Beyond providing a mere physical or steric barrier, the unique chemical architecture of KGM plays an active role in the synthesis. The polysaccharide backbone contains abundant hydroxyl groups that can coordinate with cerium ions during nucleation and assist in the partial reduction of Ce^4+^ to Ce^3+^. Consequently, KGM does not merely act as a physical barrier but also participates in the redox regulation of the Surface Ce^3+^/Ce^4+^ ratio, driving the structural and electronic optimization of the photocatalyst.

This approach aims to develop an environmentally friendly and highly efficient method for wastewater remediation, addressing critical concerns regarding dye contaminant removal from industrial effluents, and a more realistic approach to understanding the material efficiency in photocatalytic degradation. To the best of our knowledge, there has been no report using KGM as a capping and reducing agent for the synthesis of CeO_2_ NPs and reports on NBB photocatalytic degradation using CeO_2_ NPs to date.

## 2. Materials and Methods

### 2.1. Materials

Cerium Nitrate Hexahydrate (CeNO_3_·6H_2_O) ≥ 99.99% (Trace metals basis) Analytical Grade/ACS Reagent (Sigma-Aldrich, St. Louis, MO, USA), Konjac glucomannan ((KGM), Nutrigood, Santiago, Chile (Typically ≥95% glucomannan content), Hydrochloric acid (HCl) 37% (Aqueous solution (EMSURE^®^, Merck KGaA, Darmstadt, Germany), Sodium hydroxide (NaOH) ≥ 99.0% (*Pellets*) (EMSURE^®^, Merck KGaA, Darmstadt, Germany), Methyl Orange (MO) Dye content ≥ 85%, (Sigma-Aldrich, St. Louis, MO, USA), Naphthol Blue black dyes (NBB) Dye content ≥ 80%, (Sigma-Aldrich, St. Louis, MO, USA). All chemicals were purchased from Sigma-Aldrich (St. Louis, MO, USA) and Merck KGaA (Darmstadt, Germany) through their authorized distributor in Chile and were used without further purification. Double-distilled water was used throughout the experimentation.

### 2.2. Synthesis of KGM-Capped Cerium Oxide Nanoparticles (KGM-CeO_2_ NPs)

In a typical synthesis, 0.1 g of Konjac glucomannan (KGM) was dissolved in 50 mL of double-distilled water in a 250 mL round-bottom flask. The solution was stirred at room temperature until a homogeneous solution was obtained. Subsequently, 0.15 M cerium nitrate hexahydrate was added to the KGM solution, and the temperature of the reaction mixture was increased to 30 or 70 °C under constant stirring for 30 min using a hot plate magnetic stirrer. Upon the addition of the cerium salt, the color of the reaction mixture changed to white. The pH of the reaction medium was then adjusted to 9 or 11 by the slow addition of 0.625 M NaOH. During this process, the color of the reaction mixture gradually changed from white to purple and finally to pale yellow, indicating the formation of CeO_2_ nanoparticles.

The reaction mixture was then cooled to room temperature and allowed to age overnight. The resulting suspension was filtered using a filtration setup equipped with a 0.22 µm nylon membrane. The collected precipitate was washed several times with double-distilled water to remove residual impurities. The obtained nanoparticles were then dried in an oven at 55 °C and subsequently ground using a mortar and pestle until a fine powder was obtained. The resulting KGM-capped CeO_2_ nanoparticles were designated as as-synthesized KGM-CeO_2_ NPs and stored in a vacuum desiccator until further use. A portion of the as-synthesized nanoparticles was further calcined in a muffle furnace at 400 °C for 4 h and was designated as Calcinated CeO_2_ NPs.

### 2.3. Optimization of Reaction Conditions

The reaction conditions for the synthesis of KGM–CeO_2_ nanoparticles were optimized by systematically varying the pH (9–11), temperature (30–70 °C), and reaction time (4, 6, and 12 h), while keeping the concentration of KGM (0.1 g) and the precursor salt Ce(NO_3_)_3_ (0.15 M) constant. These parameters were selected to evaluate their influence on nanoparticle formation, structural properties, and overall photocatalytic performance.

### 2.4. Characterization of the Synthesized KGM-CeO_2_ Nanoparticles

As-synthesized cerium nanoparticles and calcined CeO_2_ NPs were characterized using various spectral and thermal techniques. The formation of green-synthesized CeO_2_ NPs was confirmed by using a Synergy HTX Multi-Mode reader (BioTek Instruments, Winooski, VT, USA), in a spectral scan mode with a wavelength range of 200–900 nm. The energy band gap plot was derived from the Tauc plot. KGM as a capping agent and the changes in the functional groups involved in the reduction reaction and formation of CeO_2_ NPs were examined using FTIR analysis using a CARY 630 FTIR (Agilent Technologie, Santa Clara, CA, USA), with a fixed spectral range of 4000–400 cm^−1^. The microstructure, surface morphology and mean diameter of the prepared CeO_2_ NPs were analyzed using a high-resolution transmission electron microscope (TEM) JEOL 1200 EXII, (JEOL Ltd., Tokyo, Japan). Field emission scanning electron microscopy coupled with energy-dispersive X-ray spectroscopy (FE-SEM/EDX) was performed using a JSM-7600F instrument (JEOL Ltd., Tokyo, Japan) for the determination of the morphology and for the confirmation of elements. The size of the NPS and its potential zeta were determined using a Nicomp Dynamic Light Scattering system (Particle Sizing Systems, Port Richey, FL, USA). The samples were prepared by dispersing nanoparticles in milliQ water and were analyzed for Particle size and Zeta potential. To study the crystal structure and average crystallite size of as-synthesized CeO_2_ NPs and calcinated CeO_2_ Nps, X-ray diffraction (XRD) analysis was performed using a Bruker D8 Advance X-ray diffractometer (Bruker AXS GmbH, Karlsruhe, Germany) equipped with TOPAS version 4.2 software (Bruker AXS GmbH, Karlsruhe, Germany). Diffraction patterns were recorded over a 2θ range of 20–70° using Cu Kα radiation (λ = 1.5406 Å) at room temperature. The Debye–Scherrer equation was used to calculate the average crystallite size of the catalysts.

(1) $$\tau =\frac{\;k\;\lambda \;}{\beta }cos\theta$$

where *τ* is the mean crystallite size, *K* is a dimensionless form factor (in this work, the value of 0.9 was used), *λ* is the wavelength of the incident X-rays, *β* is the full width at half maximum (FWHM), and *θ* is the diffraction angle. The average particle size has been estimated using the Debye–Scherrer equation by choosing the strongest peak arising from reflections from (111) plane at 2*θ* = ~28.5.

### 2.5. Adsorption and Photocatalytic Degradation Under UV-Light Performance of NBB, MO, and Mixed Dye Systems

The adsorption and photocatalytic performance of KGM-CeO_2_ nanoparticles (NPs) were evaluated using Naphthol Blue Black (NBB), Methyl Orange (MO), and mixed dye (NBB-MO) aqueous solutions as model organic pollutants in dark and UV light exposure. Batch experiments were conducted using 50 mL dye solutions at initial concentrations of 10, 30, 50, and 100 mg L^−1^ under acidic, neutral, and basic pH conditions to determine the optimal operational parameters for maximum dye removal efficiency.

To optimize adsorbent dosage, different amounts of KGM-CeO_2_ NPs (5, 10, and 50 mg) were added separately to each dye solution and stirred in darkness for 30 min using anSK-O330-Pro Digital Orbital Shaker (Scilogex LLC, Rocky Hill, CT, USA) to establish adsorption–desorption equilibrium and evaluate its adsorption capacity. During this stage, 800 µL aliquots were collected at 5 min intervals. Following dark equilibration, the suspensions were exposed to UV irradiation using a 40 W UV lamp(Blacklight Blue CFL, Philips, Amsterdam, The Netherlands) under continuous shaking for 120 min to assess photocatalytic degradation performance. During UV exposure, 800 µL aliquots were withdrawn every 15 min for analysis.

The collected samples were centrifuged to remove suspended nanoparticles, and the residual dye concentration was monitored using a Synergy HTX Multi-Mode reader (BioTek Instruments, Winooski, VT, USA) by measuring the characteristic maximum absorption wavelength (λ_max_) for NBB at 619 nm and 464 nm for MO, and the simultaneous absorbance of the mixed dye system was recorded at 506 nm (MO) and 619 nm (NBB) to monitor the respective degradation profiles. The degradation efficiency (%) was calculated based on the change in dye concentration before and after treatment according to the following Equation (2):

(2) $$\%\;\mathrm{Degradation}=\frac{C_{0}-C_{t}}{C_{0}}\times 100$$

where C_0_ (mg L^−1^) is the initial dye concentration, C_t_ (mg L^−1^) is the remaining dye concentration after adsorption at time t.

The adsorption capacity at equilibrium (q_e_, mg g^−1^) and at time t (q_t_, mg g^−1^) were calculated using the following Equations (3) and (4):

(3) $$q_{e}=\frac{C_{0}-C_{e}}{m}\times V$$

(4) $$q_{t}=\frac{C_{0}-C_{t}}{m}\times V$$

where C_e_ is the equilibrium dye concentration (mg L^−1^) after reaching equilibrium and V is the dye solution volume (L), and m is the mass of the CeO_2_ NPs as adsorbent (g).

To further investigate adsorption mechanisms, pseudo-first-order, pseudo-second-order, and intraparticle diffusion kinetic models were applied to the obtained experimental data. All experiments were performed in triplicate, and average values were used for analysis. This methodology enables systematic evaluation of the adsorption and photocatalytic efficiency of KGM-CeO_2_ nanoparticles toward single and mixed dye systems under varying environmental conditions.

## 3. Results and Discussion

### 3.1. Synthesis of KGM-CeO_2_ NPs

Cerium nanoparticles were synthesized under varying pH conditions (9 and 11) and temperatures (30 and 70 °C). During the synthesis, the pH of the reaction mixture was adjusted to 9 with NaOH solution, which resulted in a noticeable color change from purple to pale yellow by the end of the reaction, indicating the progression of the reaction. The as-synthesized nanoparticles were then subjected to heat treatment at 400 °C for 4 h, resulting in the formation of a yellow powder, which is characteristic of cerium oxide (ceria), confirming the successful synthesis of cerium nanoparticles. Similar results were obtained using biopolymers as a capping and stabilizing agent [35,48].

### 3.2. Characterization of KGM-CeO_2_ NPs

#### 3.2.1. UV-Visible Characterization

**Figure 1:**
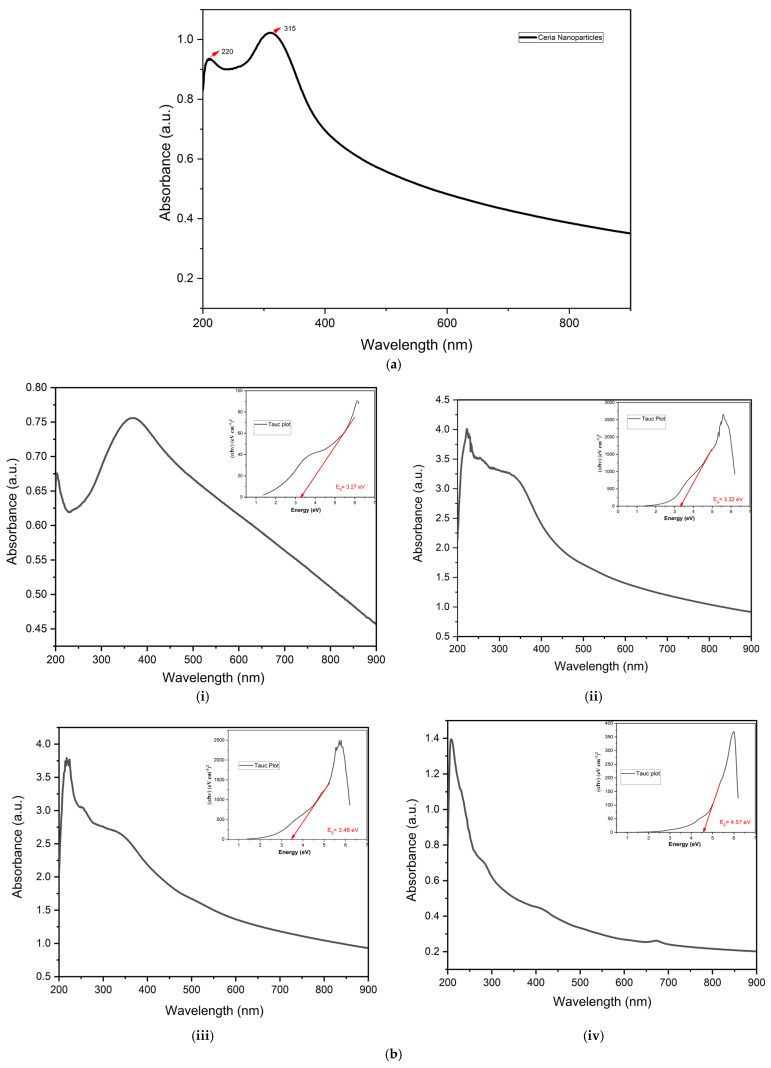
(**a**) UV–visible absorption spectra of KGM–CeO_2_ nanoparticles synthesized at pH 9 and 30 °C. (**b**) Tauc plots and corresponding optical band gap (Eg) values of KGM-CeO_2_ nanoparticles synthesized under different reaction conditions: (**i**) pH 9, 30 °C (Eg = 3.27 eV), (**ii**) pH 9, 70 °C (Eg = 3.32 eV), (**iii**) pH 11, 30 °C (Eg = 3.48 eV), and (**iv**) pH 11, 70 °C (Eg = 4.57 eV).

The optical properties of the synthesized cerium oxide nanoparticles were investigated using UV-visible absorption spectroscopy in the wavelength range of 220–900 nm, as depicted in Figure 1a. The spectra exhibited two characteristic absorption bands centered at approximately 220 nm and 315 nm, particularly for the sample synthesized at pH 9 and 30 °C. The absorption band near 220 nm is attributed to the ligand-to-metal charge transfer transition from O^2−^ 2p orbitals to Ce^4+^ states in the ceria lattice, confirming the formation of Ce–O bonds characteristic of crystalline CeO_2_. The broader absorption band around 315 nm is associated with defect-related electronic transitions arising from oxygen vacancies and the coexistence of Ce^3+^/Ce^4+^ species on the nanoparticle surface. These defect states introduce intermediate energy levels within the band structure and contribute to the enhanced redox and photocatalytic properties of ceria.

The observed absorption bands are blue-shifted compared with bulk CeO_2_ and commercial cerium oxide nanoparticles, which typically exhibit absorption maxima around 337 nm. This shift indicates the formation of smaller nanoparticles and reflects the influence of the KGM capping matrix on particle growth. Similar absorption features have been reported for green-synthesized CeO_2_ nanoparticles using *Gloriosa superba* L. and *Trianthema portulacastrum*, with absorption maxima at 297 and 292 nm, respectively [49,50]. The blue shift is attributed to the combined effects of reduced particle size, surface defects, and the stabilizing action of KGM, which controls nanoparticle nucleation and growth.

The optical band gap energies of the synthesized nanoparticles were estimated using the Tauc relationship, assuming a direct electronic transition [12], as illustrated in Figure 1b. The calculated band gap values were 3.27, 3.32, 3.48, and 4.57 eV for samples synthesized at pH 9 and 11 under temperatures of 30 and 70 °C, respectively. These values are higher than the band gap of bulk CeO_2_ (3.19 eV), indicating significant nanoscale effects. The sample synthesized at pH 9 and 30 °C exhibited the lowest band gap (3.27 eV) and the smallest crystallite size (7.77 nm), while increasing pH and temperature resulted in larger crystallite sizes and corresponding changes in the optical response. The CeO_2_ particle size increased from 8.28 to 11.83 nm under higher pH and temperature conditions, demonstrating the influence of synthesis parameters on nanoparticle growth and optical properties. Similar band-gap widening has been reported for green-synthesized CeO_2_ nanoparticles, with values ranging from 3.44 to 3.70 eV [51].

A low-energy shoulder was observed in the Tauc plots, which can be attributed to defect states and oxygen vacancies within the ceria lattice. These defect-induced states create localized energy levels within the band gap and influence the optical absorption behavior. Therefore, the observed variations in band gap energy are associated not only with changes in crystallite size but also with differences in defect concentration, oxygen vacancy density, and the relative Ce^3+^/Ce^4+^ ratio. Furthermore, the KGM matrix helps maintain the nanoparticles in a porous and well-dispersed structure, preserving their nanoscale characteristics, increasing the accessible surface area, and enhancing their adsorption and photocatalytic performance toward NBB and MO dye degradation.

#### 3.2.2. FT-IR

**Figure 2:**
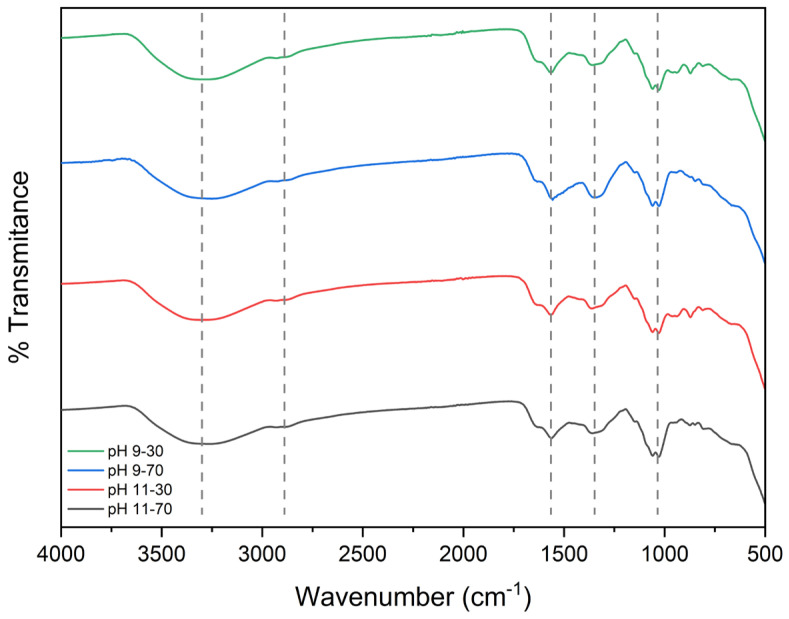
ATR–FTIR spectrum of KGM–CeO_2_ nanoparticles synthesized under different pH (9 and 11) and temperature (30 and 70 °C) conditions. The vertical dashed lines indicate the key analytical coordinates tracked across samples at 3300 cm^−1^ (O–H stretching), 2890 cm^−1^ (C–H stretching), 1565 cm^−1^ (C=O/O–H bending), 1348 cm^−1^ (CO_3_^2−^ surface carbonates), and 1035 cm^−1^ (C–O–C) glycosidic linkage.

Figure 2 shows the ATR–FTIR spectrum of KGM–CeO_2_ nanoparticles synthesized under different reaction conditions (pH 9–11 and temperatures of 30–70 °C). The spectra exhibited similar characteristic functional group regions with noticeable variations in peak intensity, suggesting differences in nanoparticle crystallinity, surface interaction, and capping efficiency under varying synthesis conditions. The broad absorption bands observed around 3500–2800 cm^−1^ (centred at 3300 cm^−1^), including peaks at 3315–3345 cm^−1^, are attributed to the stretching vibration of hydroxyl (O–H) groups originating from the KGM polysaccharide backbone and adsorbed water molecules associated with the CeO_2_ nanoparticle surface [52]. Weak absorption bands detected at 2890 cm^−1^ are assigned to aliphatic C–H stretching vibrations, confirming the presence of the organic biopolymer matrix. A well defined absorption band centered at 1565 cm^−1^ corresponds to the bending vibration of adsorbed water molecules [δ(OH)], and it may also indicate contributions from carbonyl-related interactions of acetyl groups present in KGM, where the noticeable variations in symmetry and intensity between pH 9 and pH 11 confirm the alkaline-induced partial de-acetylation of the framework. Furthermore, the sharp peaks observed at 1348 cm^−1^ are associated with carbonate species adsorbed on the CeO_2_ surface, while the band around 1035 cm^−1^ corresponds to C–O–C glycosidic stretching vibrations characteristic of the KGM structure, further confirming successful stabilization and capping of the nanoparticles by the biopolymer. Crucially, the sample synthesized at pH 11 and 30 °C exhibits uniquely sharp, well-defined skeletal peaks near 1035 cm^−1^, indicating that the combination of high alkalinity and low thermal energy induces the de-acetylated biopolymer chains to condense into dense, highly ordered localized polymeric pockets. This structural phenomenon directly restrict chain expansion and entrap nascent ceria nuclei in close spatial proximity, driving aggregation-mediated crystalline fusion into larger, highly crystalline grains before uniform steric capping can be established. Conversely, comparative analysis of the spectra revealed that the samples synthesized at pH 11 and 70 °C exhibited stronger and sharper characteristic peaks across the entire profile, indicating that the elevated thermal energy yields an effective, uniformly distributed steric barrier that prevents aggregation, successfully isolating individual nanoparticles, improving crystallinity, and leading to more effective nanoparticle stabilization. Finally, the characteristic absorption bands appearing in the low-frequency fingerprint region below 850 cm^−1^ are attributed to metal–oxygen stretching vibrations (Ce–O) and characteristic CeO_2_ phonon modes, providing clear evidence for the successful formation of CeO_2_ NPs within the KGM matrix [53].

#### 3.2.3. XRD

**Figure 3:**
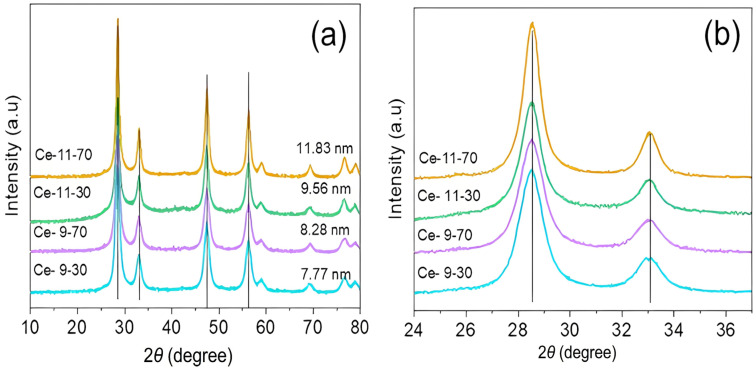
(**a**) XRD patterns of KGM–CeO_2_ nanoparticles synthesized under different reaction conditions: (i) pH 9, 30 °C; (ii) pH 9, 70 °C; (iii) pH 11, 30 °C; and (iv) pH 11, 70 °C. (**b**) Enlarged view of the diffraction peaks highlighting the progressive increase in crystallinity and crystallite size with increasing pH and reaction temperature.

Figure 3a displays X-ray diffraction patterns of various KGM-CeO_2_ NPs samples. The crystallinity and particle size vary with the reaction conditions. At pH 9, the samples prepared at 30° and 70 °C exhibit an average particle size of 7.77 and 8.28 nm, respectively. Similarly, slightly larger average particle sizes were obtained at pH 11 with an average particle size of 9.56 and 11.83 at 30 and 70 °C. Both the crystallinity and particle size increase with pH and reaction temperature. These slight variations in the nature of ceria nanoparticles are more clearly observed in the magnified XRD Figure 3b [7].

#### 3.2.4. SEM

**Figure 4:**
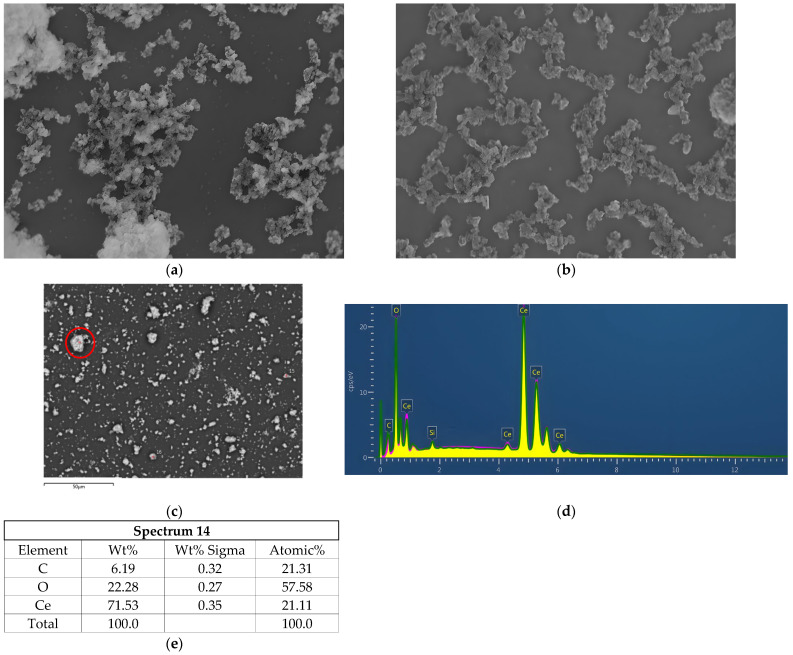
FESEM micrographs (**a**–**c**) and corresponding EDX analysis profiles of the synthesized KGM–CeO_2_ NPs. Panels (**a**–**c**) the porous, sponge-like morphological features under increasing magnifications, where the red circle annotated in panel (**c**) denotes the localized spot target selected for microanalysis. Panel (**d**) presents the resolved EDX elemental spectrum captured from this targeted region, and table (**e**) lists the corresponding quantitative elemental composition by weight and atomic percentage.

The SEM analysis revealed the formation of irregular quasi-spherical cerium nanoparticles with a porous, sponge-like morphology. As shown in Figure 4a,b the nanoparticles prepared at pH 9 at 30° C exhibit a refined porous structure with an interconnected and branched network, resulting in a high degree of surface area exposure. Such morphology is characteristic of nanoparticles synthesized using biopolymer templates such as KGM. Furthermore, the sponge-like porous architecture, as shown in Figure 4a is attributed to the capping effect of KGM, which minimizes severe agglomeration and promotes the formation of a three-dimensional framework with abundant accessible surface sites. This characteristic morphology is highly beneficial for adsorption and photocatalytic applications, where the inter-particle voids act as diffusion channels for NBB and MO dye molecules, thereby enhancing dye adsorption and improving photocatalytic degradation efficiency under UV light irradiation [9,54]. Similar porous and interconnected morphologies were also observed for KGM-CeO_2_ nanoparticles synthesized under different pH and temperature conditions. However, variations in synthesis parameters resulted in changes in particle uniformity, with some samples exhibiting more irregularly shaped particles and a higher degree of agglomeration. These differences may be associated with changes in nucleation and growth rates during synthesis, which influence particle stabilization by the biopolymeric KGM matrix.

The EDS spectrum shown in Figure 4d, corresponding to the spot selected in the SEM Figure 4c indicated by the red circle, confirms the successful formation of CeO_2_ nanoparticles with high purity. The elemental analysis revealed the presence of Ce and O as the major constituents, with weight percentages of 71.53% and 22.28%, respectively, confirming the characteristic composition of cerium oxide nanoparticles. In addition, a noticeable carbon content (6.19 wt%) was observed, which can be attributed to the presence of the carbon during the sample preparation. The strong cerium peaks observed in the EDS spectrum demonstrate the high crystallinity and successful incorporation of cerium within the synthesized material, while the oxygen peak confirms the oxide nature of the nanoparticles. The homogeneous elemental distribution as shown in Figure 4e it demonstrated absence of significant impurity peaks, suggesting that the synthesis process produced relatively pure CeO_2_ nanoparticles. Furthermore, the carbon-rich surface may contribute to improved dispersion and reduced agglomeration, enhancing the adsorption behavior and photocatalytic efficiency of the nanoparticles toward dye degradation applications.

#### 3.2.5. TEM

**Figure 5:**
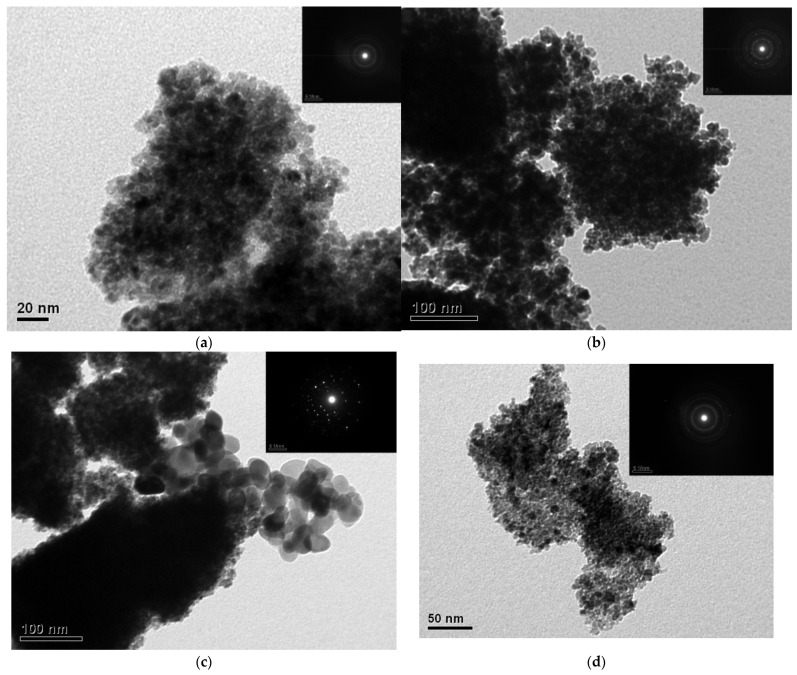
TEM images of KGM–CeO_2_ nanoparticles synthesized under different reaction conditions: (**a**) pH 9, 30 °C; (**b**) pH 9, 70 °C; (**c**) pH 11, 30 °C; and (**d**) pH 11, 70 °C.

Figure 5 shows the TEM images of various KGM-CeO_2_ samples. The nature of samples is strongly influenced by reaction conditions. Figure 5a,b reveal the amorphous nature of ceria. The digital diffraction patterns show the presence of rings and absence of spots, indicating the presence of a poorly crystalline nature. Figure 5c exhibits the co-presence of rings and spots, indicating a polycrystalline nature. This localized grain growth at 30 °C and pH 11 is attributed to the alkaline-induced deacetylation and subsequent aggregation of KGM chains into dense polymeric pockets at low thermal energy. These pockets entrap nascent ceria nuclei in proximity, promoting aggregation-mediated crystalline fusion before full steric capping is achieved. Conversely, as the reaction temperature increases from 30 to 70 °C, the high kinetic energy of the KGM chains provides an effective and uniform steric barrier that prevents aggregation, successfully isolating individual nanoparticles and keeping them small. Therefore, the material in Figure 5c consists of nanocrystalline CeO_2_ cores embedded within or coated by an amorphous KGM matrix, resulting in the coexistence of crystalline and amorphous characteristics. In Figure 5d, the diffraction pattern shows rings with spots, indicating the crystallinity [49,52,55]. These results are in good corroboration with the XRD results.

#### 3.2.6. DLS and ZP

Dynamic light scattering (DLS) studies by GAUSSIAN/NICOMP distribution Analysis revealed, as shown in Figure S1, Nicomp distribution exhibited a bimodal particle size distribution for KGM-CeO_2_ NPs synthesized at pH 9 and 30 °C, indicating the coexistence of a dominant nanoscale particle population and a secondary aggregated fraction. Volume-weighted NICOMP analysis revealed that the principal nanoparticle population was centered at approximately 29.7 nm, accounting for 72.67% of the total particle volume, while a secondary broader population at approximately 209.7 nm represented 27.33%, suggesting moderate aggregation of polymer-coated nanoparticles in suspension. Similarly, number-weighted analysis showed that the majority of particles (99.11%) were distributed around 28.8 nm, confirming that the synthesized nanoparticles predominantly exist within the nanoscale range.

The Gaussian distribution further supported this observation, with a number-weighted mean diameter of 111.2 nm and an intensity-weighted mean diameter of 217.9 nm, reflecting the greater scattering contribution of larger aggregated structures. The presence of the larger hydrodynamic fraction is likely associated with partial interparticle association, hydration layers, and the konjac glucomannan (KGM) biopolymer shell surrounding the CeO_2_ nanocrystals. Such behavior is expected in polysaccharide-capped metal oxide systems, where the stabilizing polymer matrix enhances aqueous dispersibility but may also promote soft agglomeration through intermolecular interactions [56,57].

When compared with XRD-derived crystallite sizes (~7–9 nm), the larger DLS/NICOMP dimensions indicate that individual CeO_2_ nanocrystals are assembled into larger polymer-stabilized colloidal nanoparticles rather than existing as isolated crystals. This hierarchical nanostructure is advantageous for adsorption and photocatalytic applications because the small crystalline core preserves high surface reactivity, while the KGM coating improves colloidal stability, functional group availability, and pollutant interaction. Overall, the DLS/NICOMP results confirm the successful synthesis of predominantly nanosized KGM-CeO_2_ nanoparticles with moderate aggregation behavior, supporting their suitability for efficient dye adsorption and photocatalytic degradation applications.

#### 3.2.7. Zeta Potential Analysis

The surface charge and colloidal stability of the KGM-CeO_2_ nanoparticles prepared at pH 9 at 30 °C were evaluated by electrophoretic light scattering using a Particle Sizing Systems Zeta Potential Analyzer at 23 °C, without pH adjustment under an applied electric field of 10 V/cm. It demonstrated that the average electrophoretic mobility was 1.25 µm·cm/V·s, and from Figure S2, it can be observed that the mean zeta potential was +16.75 mV. The zeta potential measurements revealed that the KGM-CeO_2_ nanoparticles synthesized at pH 9 and 30 °C exhibited the highest positive surface charge (+16.75 mV) compared to the samples synthesized at pH 9 and 70 °C (+9.65 mV), pH 11 and 30 °C (−7.48 mV), and pH 11 and 70 °C (−12.90 mV). This higher positive zeta potential indicates improved colloidal stability and a greater degree of surface functionalization by KGM. Although the measured value is below the ±30 mV threshold generally associated with highly stable dispersions, the polymeric KGM coating likely provides additional steric stabilization, reducing nanoparticle aggregation. The sample synthesized at pH 9 and 30 °C was selected for further adsorption and photocatalytic studies because it combined the highest positive surface charge, the best colloidal stability, the smallest crystallite size, and superior photocatalytic performance. Moreover, the positive surface charge favors stronger electrostatic attraction toward negatively charged dye molecules, enhancing adsorption efficiency and facilitating subsequent photocatalytic degradation. In contrast, the samples synthesized at pH 11 exhibited negative zeta potential values, which are less favorable for the adsorption of anionic dyes due to electrostatic repulsion. Therefore, the zeta potential results support the selection of the pH 9, 30 °C sample as the most suitable candidate for photocatalytic applications.

### 3.3. Adsorption and Photocatalytic Degradation of NBB, MO, and Mixed Dyes Under UV Light by KGM-CeO_2_NPs

#### 3.3.1. Optimization of Adsorbent Concentration for NBB Removal

**Figure 6:**
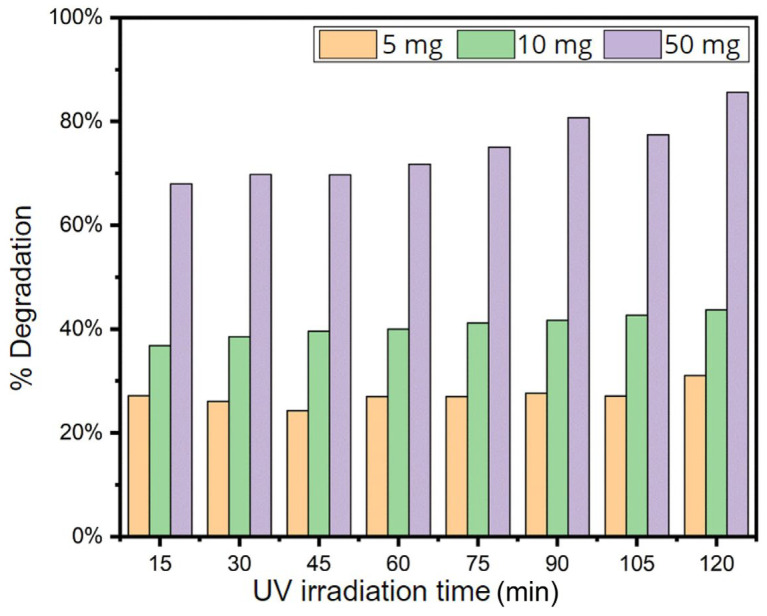
Photocatalytic degradation efficiency of Naphthol Blue Black dye under UV irradiation using different dosages of KGM–CeO_2_ nanoparticles.

NBB is highly resistant to self-photolysis and exhibits relatively slow degradation under visible light due to its stable aromatic azo structure and strong resistance to oxidative breakdown. Therefore, effective degradation of this dye requires the combined action of an adsorbent material and UV irradiation. The degradation efficiency strongly depends on the amount of adsorbent used, since increasing the nanoparticle dosage enhances the availability of active surface sites for dye adsorption. The initial adsorption–photocatalytic experiments were conducted to determine the optimal KGM–CeO_2_ nanoparticle dosage required for efficient NBB degradation under UV irradiation. Three adsorbent concentrations corresponding to 5, 10, and 50 mg, respectively, were evaluated. As shown in Figure 6, increasing nanoparticle concentration significantly enhanced NBB degradation efficiency, confirming the concentration-dependent availability of active adsorption and photocatalytic sites. Among the tested dosages, a concentration of 50 mg exhibited the highest performance, achieving 85.65% degradation after 120 min of UV exposure, whereas concentrations of 5 and 10 mg resulted in 31.06% and 43.72% degradation, respectively. This enhanced degradation at higher adsorbent dosage can be attributed to the greater surface area, increased number of active adsorption sites, and improved photon-induced catalytic interactions provided by the larger quantity of nanoparticles. Therefore, 50 mg was selected as the optimal adsorbent concentration for subsequent experiments.

#### 3.3.2. Effect of pH on NBB Adsorption and Photocatalytic Degradation

**Figure 7:**
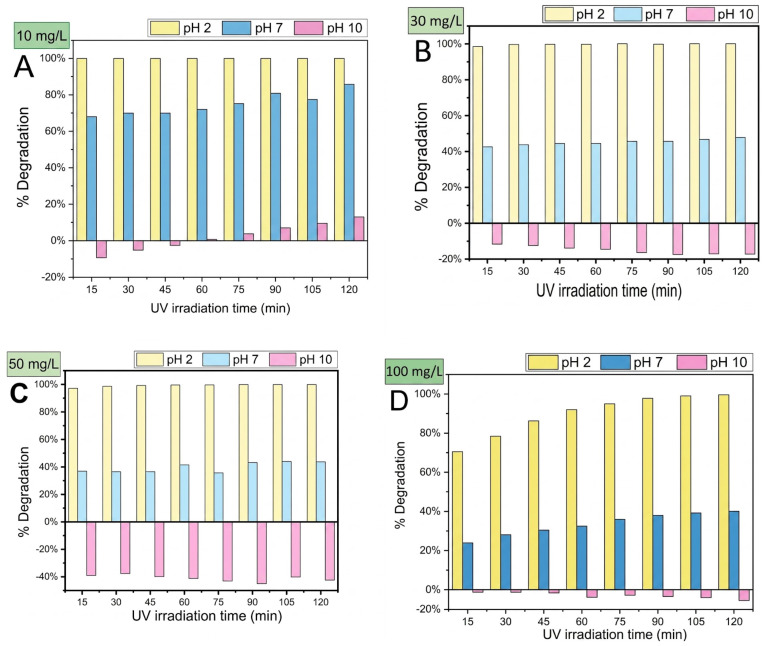
Photocatalytic degradation profiles of Naphthol Blue Black (NBB) under UV irradiation at different pH conditions and dye concentrations: (**A**) 10 mg/L, (**B**) 30 mg/L, (**C**) 50 mg/L, and (**D**) 100 mg/L.

Solution pH played a critical role in NBB degradation efficiency across all dye concentrations (10–100 mg/L). Using the optimized adsorbent dosage (50 mg), degradation experiments under acidic, natural, and basic conditions demonstrated that acidic pH consistently provided the highest removal efficiency (Figure 7). NBB is an anionic dye due to the presence of sulfonate and carboxylate functional groups in its molecular structure. At acidic pH = 2, degradation efficiencies exceeded 99% for all concentrations, reaching 99.79%, 99.97%, 99.93%, and 99.41% for 10, 30, 50, and 100 mg/L, respectively. The enhanced degradation observed under acidic conditions can be attributed to the positively charged surface of the photocatalyst, which promotes electrostatic attraction between the catalyst surface and the negatively charged dye molecules. Furthermore, acidic media favor the formation of reactive oxygen species, particularly hydroxyl radicals (•OH), which possess strong oxidative potential and are mainly responsible for breaking azo bonds (-N=N-), aromatic rings, and other chromophoric groups present in the dye structure. Consequently, both adsorption and subsequent oxidation/mineralization of NBB are significantly enhanced.

In contrast, natural pH = 7 resulted in moderate degradation (39.98–85.65%), which may be attributed to the reduced photocatalytic efficiency caused by the excess hydroxide ions (OH^−^) present in solution. While basic pH = 10 produced negligible or even negative degradation values, this may be due to the catalyst surface, which tends to become negatively charged, resulting in electrostatic repulsion between the catalyst and the anionic dye molecules, thereby hindering effective adsorption onto the nanoparticle surface. In addition, the excessive OH^−^ concentration may promote electron-hole recombination, reducing the generation of oxidative radicals and consequently decreasing photocatalytic degradation efficiency. The negative degradation values may also indicate dye instability or possible spectral interference under alkaline conditions.

The superior performance under acidic conditions at pH = 2 is likely due to protonation of functional groups on the KGM–CeO_2_ surface, enhancing electrostatic attraction between positively charged adsorbent sites and the anionic sulfonate groups of NBB. Furthermore, acidic conditions may facilitate improved charge separation and reactive oxygen species generation during UV irradiation, promoting photocatalytic degradation. The poor performance under basic conditions may be associated with electrostatic repulsion, hydroxyl ion competition, and reduced catalytic efficiency.

**Figure 8:**
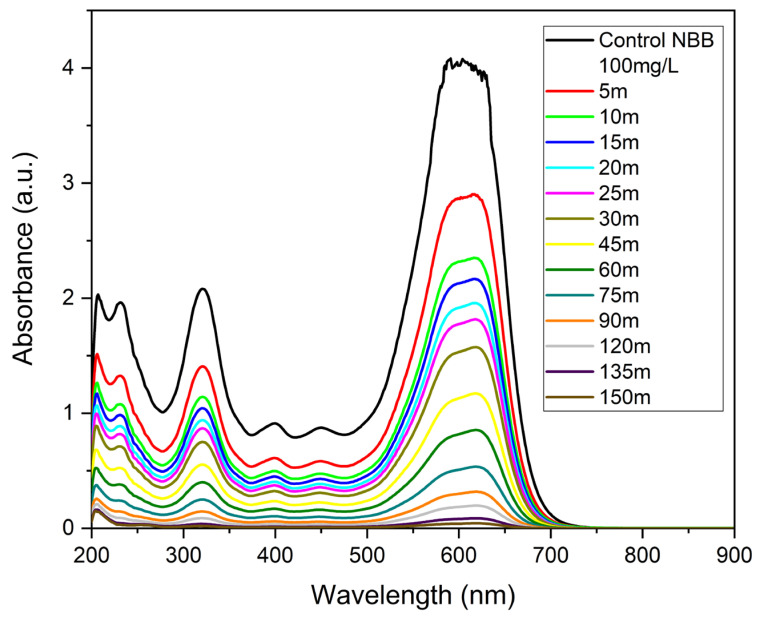
UV–Vis absorbance changes of Naphthol Blue Black dye (NBB) during photocatalytic degradation under acidic conditions (pH 2) at an initial dye concentration of 100 mg/L.

UV–Vis spectral analysis of NBB degradation at 100 mg/L under acidic conditions, as shown in Figure 8, further confirmed efficient molecular breakdown. Major absorbance peaks at 230, 320, 399, 450, and 619 nm were progressively reduced over time, indicating destruction of chromophoric and aromatic structures, while the persistence of the 210 nm peak suggests the possible formation of low-molecular-weight intermediates.

**Figure 9:**
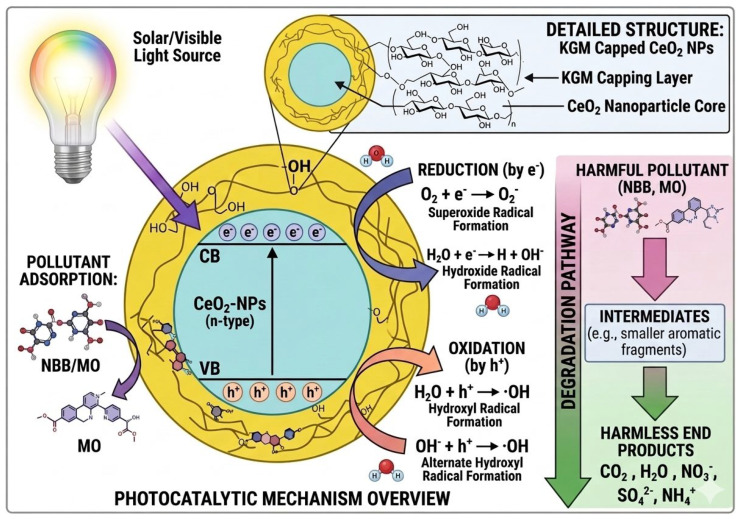
Proposed mechanism for the photocatalytic degradation of azo dyes, including Naphthol Blue Black and Methyl Orange, using KGM–CeO_2_ nanoparticles under UV irradiation.

The photocatalytic mechanism under UV irradiation is illustrated in Figure 9, while foundational photocatalysis occurs via electronic band-gap excitation of the CeO_2_ core, the surrounding KGM matrix enhances degradation through distinct surface phenomena. First, hydrophilic KGM acts as a molecular sponge that pre-concentrates anionic dyes via electrostatic and hydrogen-bonding interactions directly at active catalytic nodes. Second, its long polymer chains exert a steric hindrance effect that prevents CeO_2_ agglomeration, preserving an ultra-small particle size and maximizing exposed active surface area. Third, the electron-dense, oxygen-rich KGM network temporarily traps photogenerated valence band holes (h^+^) at the hybrid interface. This interfacial charge stabilization retards carrier recombination, significantly extending the lifetime of conduction band electrons (e^−^) to generate a higher volume of reactive oxygen radicals.

Consequently, upon UV irradiation, electrons from the valence band (VB) absorb sufficient energy and migrate to the conduction band (CB), generating electron-hole pairs (e^−^/h^+^):

(5) $$KGM-CeO_{2}+h\nu \to {KGM-CeO_{2}(e}_{CB}^{-}+h_{VB}^{+})$$

Due to the suppressed recombination rate facilitated by the interfacial hole-trapping effect of the KGM coating, the accumulated conduction band electrons ($e_{CB}^{-}$) readily react with dissolved oxygen to produce superoxide radicals (•O_2_^−^).

(6) $$e_{CB}^{-}+O_{2}\to •O_{2}^{-}$$

Simultaneously, the stabilized, long-lived holes ($h_{VB}^{+}$) escaping the core matrix react with water molecules or surface-bound hydroxyl groups to generate highly reactive hydroxyl radicals (·OH), as shown below:

(7) $$h_{VB}^{+}+H_{2}O\to •OH+H^{+}$$

(8) $$h_{VB}^{+}+{OH}^{-}\to •OH$$

Because the target pollutants are already pre-concentrated and securely held within the hydrophilic 3D network of the KGM matrix, these reactive oxygen species (ROS) attack the adjacent, adsorbed NBB, MO, or mixed dye molecules with minimal mass transfer resistance. This immediate, close-proximity radical attack leads to the progressive cleavage of the highly stable azo bonds and complex aromatic structures, yielding smaller fragments and guiding them toward final mineralization.

•O_2_^−^ + Dye → Degradation products (9)

•OH + Dye → CO_2_ + H_2_O + mineralized products (10)

This multi-functional synergistic mechanism confirms that the KGM matrix does not act merely as an inert structural carrier, but actively participates in the enhanced, high-performance hybrid photocatalytic process compared to pristine metal oxides.

The experimental results demonstrated that, for the 100 mg/L NBB dye solution (Figure 7), the degradation percentage increased progressively during UV irradiation, indicating a significant contribution of the photocatalytic mechanism. In contrast, for lower dye concentrations (10, 30, and 50 mg/L), the highest removal efficiency occurred during the dark equilibrium stage, suggesting that adsorption onto the catalyst surface was the dominant process. This behavior indicates that at low dye concentrations, sufficient active sites are available for adsorption, whereas at higher concentrations, partial saturation of adsorption sites requires greater participation of photocatalytically generated reactive species to achieve high degradation efficiencies and mineralization of the dye molecule.

### 3.4. Adsorption and Photocatalytic Degradation of MO by KGM-CeO_2_ NPs

#### 3.4.1. Effect of pH on MO Removal

**Figure 10:**
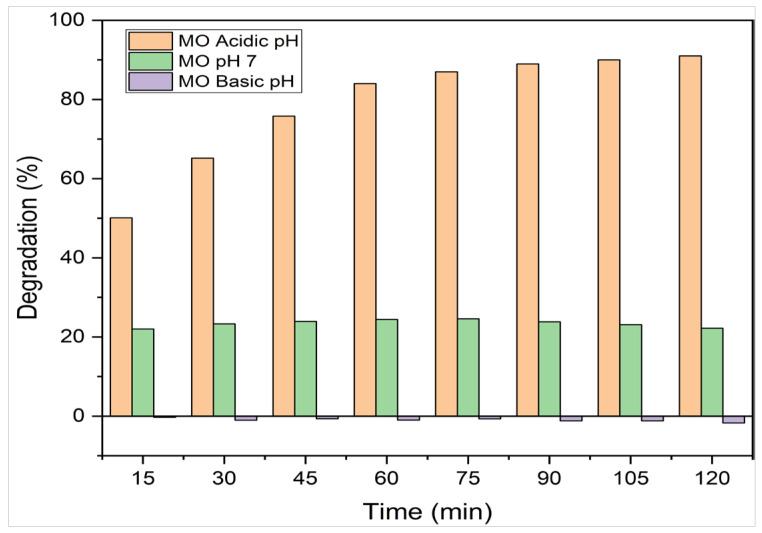
Adsorption efficiency of Methyl Orange dye onto KGM–CeO_2_ nanoparticles under different pH conditions.

Similar to NBB, MO has an azo structure and exhibits low degradation in visible light. As shown in Figure 10, MO degradation was strongly influenced by solution pH and adsorbent dosage. At an adsorbent dosage of 50 mg in acidic pH conditions, the highest degradation efficiency was reported (91%), significantly outperforming neutral (22%) and basic conditions (0%) under UV exposure for 120 min. These findings confirm that acidic conditions and higher dosage amounts are optimal for MO degradation and suggest a similar adsorption–photocatalytic mechanism to NBB. The enhanced MO degradation under acidic pH is attributed to improved electrostatic attraction between protonated KGM-CeO_2_ active sites and the anionic azo dye structure of MO. Under basic conditions, the absence of degradation suggests electrostatic repulsion and limited photocatalytic activity as observed for NBB dye.

**Figure 11:**
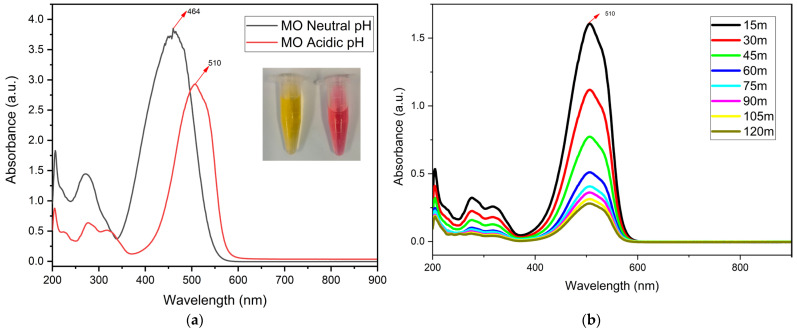
(**a**) UV visible spectra of MO with KGM-CeO_2_ nanoparticles in neutral and acidic conditions (**b**) MO photocatalytic degradation under UV irradiation for 120 min.

Furthermore, UV–Vis spectroscopy (Figure 11a) demonstrated that pH adjustment significantly influenced the optical properties of MO, producing a shift in its characteristic maximum absorption peak from 464 nm to 510 nm, which is associated with protonation of the azo dye structure under acidic conditions. This spectral shift was accompanied by a visible color change in the dye solution (Figure 11a inset), confirming alteration of the electronic environment of the chromophoric group and indicating that acidic pH favors molecular activation for subsequent adsorption and photocatalytic degradation. During the initial dark adsorption stage (0–30 min), UV–Vis spectra (Figure 11b) revealed a gradual decrease in absorbance intensity, indicating that MO molecules were effectively adsorbed onto the surface of KGM-CeO_2_ nanoparticles before UV activation. This behavior confirms the strong adsorptive affinity of the nanomaterial toward MO, likely promoted by electrostatic interactions between the positively charged nanoparticle surface and the anionic dye molecules. The progressive reduction during this stage demonstrates that adsorption serves as an important pre-concentration step, facilitating close contact between the dye molecules and active catalytic sites. Upon subsequent UV irradiation for 120 min, a pronounced and continuous decline in the absorbance intensity at 510 nm was observed (Figure 11b), confirming progressive photocatalytic degradation of MO. The marked reduction in the characteristic absorption band indicates disruption of the azo chromophore (-N=N-), which is responsible for the dye’s color and structural stability. This result suggests that UV-activated KGM-CeO_2_ nanoparticles generate reactive oxygen species capable of oxidizing and decomposing MO molecules beyond simple adsorption.

#### 3.4.2. Adsorption and Photocatalytic Degradation of MO in Varying Concentrations by KGM-CeO_2_ NPs

**Figure 12:**
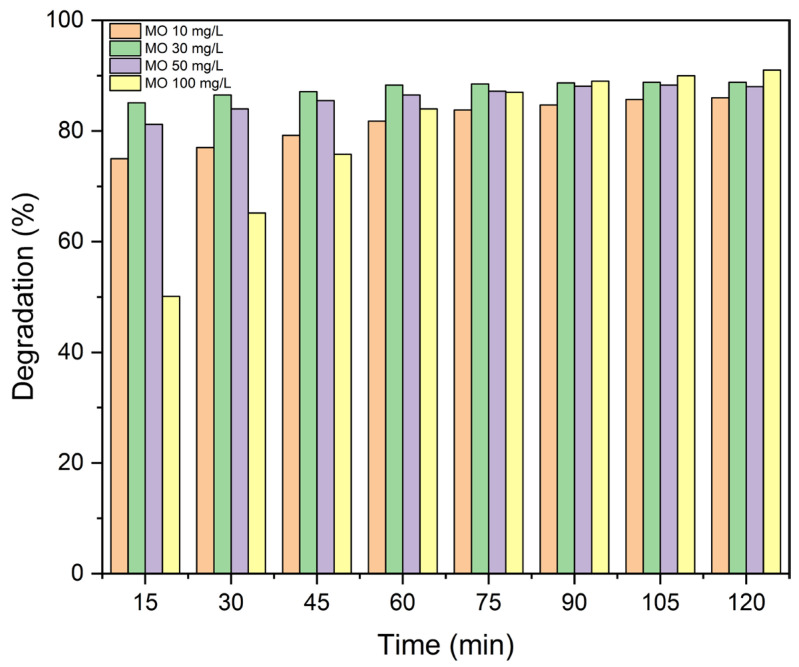
Photocatalytic degradation efficiency of Methyl Orange under UV irradiation at different dye concentrations under acidic conditions.

As shown in Figure 12, all tested MO concentrations (10, 30, 50, and 100 mg L^−1^) exhibited high degradation efficiencies under acidic conditions, achieving removal percentages above 86% after 120 min of UV exposure. Specifically, degradation efficiencies of 86%, 88%, 88%, and 91% were obtained for 10, 30, 50, and 100 mg L^−1^, respectively. The highest degradation efficiency observed at 100 mg L^−1^ indicates that KGM-CeO_2_ nanoparticles retain excellent photocatalytic activity even at elevated pollutant concentrations. This behavior may be attributed to the synergistic combination of high adsorption capacity provided by the KGM polymer matrix and the photocatalytic redox activity of CeO_2_ nanoparticles, which together sustain efficient pollutant removal under increased dye loading.

The enhanced degradation at higher concentrations also suggests that the active surface sites and photocatalytic centers of KGM-CeO_2_ were not rapidly saturated, reflecting favorable surface functionality and pollutant accessibility. And the mechanism behind the photocatalytic degradation seems to be similar as that of NBB. Overall, these findings confirm the bifunctional nature of KGM-CeO_2_ nanoparticles, where initial adsorption during dark conditions is followed by efficient photocatalytic oxidation under UV irradiation. This dual mechanism significantly improves MO removal efficiency and highlights the potential of KGM-CeO_2_ NPs as effective multifunctional platforms for wastewater remediation, particularly for azo dye-contaminated systems.

From the above studies, it was observed that acidic conditions play a crucial role in the adsorption and photocatalytic degradation of both NBB and MO. Therefore, the mixed dye system was investigated under acidic conditions using a combined dye concentration of 100 mg/L as employed for NBB and MO.

### 3.5. Adsorption and Photocatalytic Degradation of Mixed Dyes (NBB-MO) by KGM-CeO_2_

**Figure 13:**
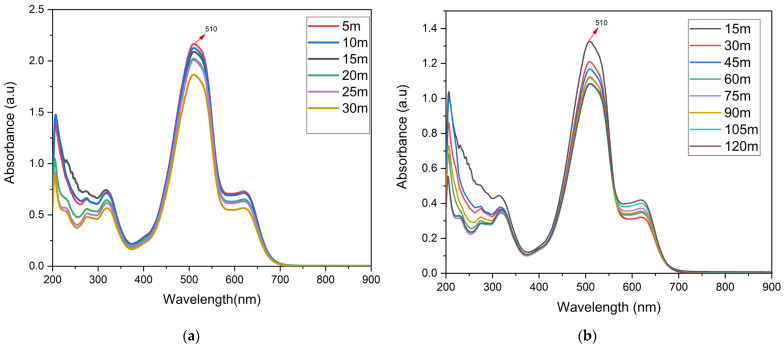
UV–Vis absorption spectra of the mixed dye system containing Naphthol Blue Black and Methyl Orange (100 mg/L) (**a**) adsorption under dark conditions and (**b**) photocatalytic degradation under UV irradiation.

As shown in the UV–Vis absorption spectra in Figure 13a, the mixed dye (NBB-MO) solution under dark conditions showed a gradual decrease in absorbance intensity from 5 to 30 min, indicating effective adsorption of the dye molecules onto the KGM–CeO2 nanoparticles surface prior to UV irradiation. The most significant decrease in absorbance occurred during the initial adsorption period, followed by stabilization of the spectra toward 25–30 min, suggesting that adsorption–desorption equilibrium was achieved. No significant shift in the absorption peaks was observed, indicating that the dye structures remained largely unchanged during the dark stage and that adsorption was the dominant removal mechanism rather than photocatalytic degradation.

The observed adsorption behavior may be attributed to electrostatic interactions, hydrogen bonding, and surface complexation between the negatively charged dye molecules and the active sites of the nanoparticles. The broad absorption bands corresponding to both dyes decreased simultaneously, demonstrating that the material possesses strong affinity toward the mixed dye system. This adsorption stage is particularly important because it facilitates closer contact between the dye molecules and the photocatalyst surface, thereby enhancing the subsequent photocatalytic degradation process under UV irradiation.

UV–Vis absorbance spectra, as shown in Figure 13b, demonstrated a gradual decrease in absorbance intensity during UV irradiation, indicating the progressive degradation of the mixed dye system. The synthesized KGM–CeO_2_ nanoparticles achieved an overall degradation efficiency of approximately 52% for the mixed NBB–MO dye solution, confirming their combined effect of surface adorption and photocatalytic activity toward multicomponent dye pollutants. Although both dyes showed excellent degradation efficiencies individually, the mixed dye system exhibited comparatively lower removal performance, indicating the increased complexity of multicomponent dye degradation systems that more closely resemble real textile wastewater. Individually, NBB generally exhibited slightly higher degradation efficiency (>99%) compared with MO (86–91%), suggesting a stronger affinity of NBB toward the KGM–CeO_2_ nanoparticles surface. This behavior may be attributed to the larger aromatic structure and the presence of multiple sulfonate groups in NBB, which can promote stronger electrostatic interactions, hydrogen bonding, π–π interactions, and surface complexation with the photocatalyst surface. In contrast, MO possesses a relatively smaller molecular structure and fewer interaction sites, which may result in comparatively weaker adsorption and photocatalytic interaction with the catalyst surface.

In the mixed dye system, both dye molecules simultaneously compete for the limited active adsorption sites available on the photocatalyst surface. Since adsorption is a critical preliminary step for efficient photocatalytic degradation, this competitive adsorption reduces the accessibility of reactive sites for each dye molecule. Furthermore, the reactive oxygen species generated during UV irradiation, particularly hydroxyl radicals (•OH) and superoxide radicals (•O^2−^), become insufficient relative to the total number of dye molecules present in solution. Consequently, the oxidative degradation efficiency decreases compared with single-dye systems. Another important factor contributing to the lower degradation efficiency is the light attenuation or shielding effect. Mixed dye solutions possess higher overall color intensity and absorb a broader range of UV radiation, thereby limiting photon penetration into the reaction medium. This reduces electron excitation within the CeO_2_ nanoparticles and decreases the generation of electron-hole pairs and reactive radicals responsible for dye degradation. Additionally, intermediate degradation products generated from one dye may adsorb onto the catalyst surface and partially block active sites, further inhibiting degradation of the second dye molecule. The degradation mechanism in the mixed dye system involves a combination of adsorption, photocatalytic oxidation, radical-mediated degradation, and competitive interactions between dye molecules. Under UV irradiation, CeO_2_ nanoparticles generate electron-hole pairs that react with dissolved oxygen and water molecules to produce reactive oxygen species capable of attacking azo bonds (-N=N-) and aromatic structures in both dyes. However, in the multicomponent system, competition for active sites and oxidative radicals slows the degradation kinetics compared with individual dye systems.

These findings are highly relevant from an industrial perspective because actual textile wastewater typically contains mixtures of dyes, salts, surfactants, and organic additives rather than single dye pollutants. Therefore, the mixed dye experiment provides a more realistic simulation of industrial effluent conditions. The obtained results demonstrate that the synthesized photocatalyst exhibits excellent efficiency for single-dye degradation and promising applicability for complex wastewater treatment systems. Nevertheless, further optimization of operational parameters, catalyst dosage, irradiation conditions, and catalyst surface properties may be necessary to improve degradation efficiency in multicomponent dye systems representative of real textile effluents.

### 3.6. Adsorption Kinetics and Mechanistic Modeling of NBB, MO, and Mixed Dye (NBB-MO) Systems

#### 3.6.1. Adsorption Kinetics of NBB

**Figure 14:**
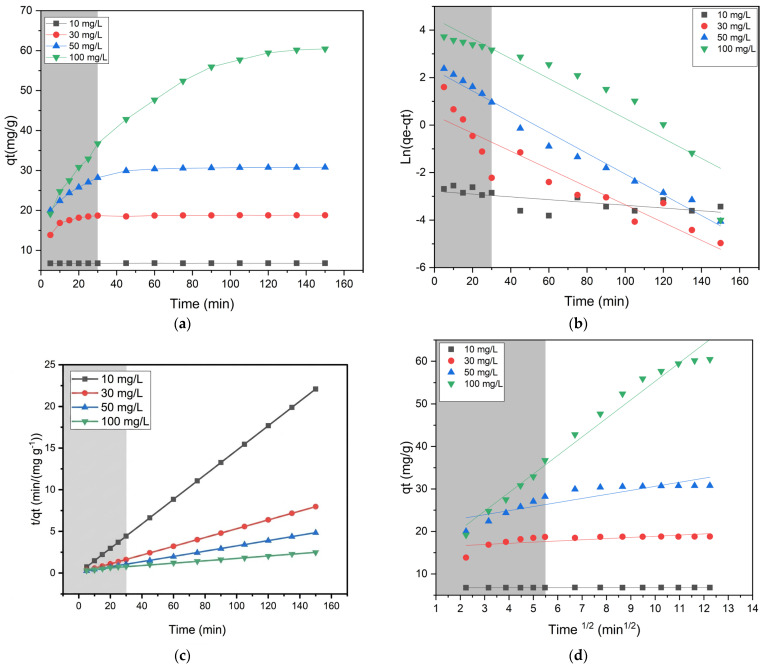
Adsorption kinetic studies for the degradation of Naphthol Blue Black onto KGM–CeO_2_ nanoparticles: (**a**) adsorption kinetics profile, (**b**) pseudo-first-order model, (**c**) pseudo-second-order model, and (**d**) intra-particle diffusion model.

The amount of NBB dye adsorbed per unit mass (qt) over time can be seen in Figure 14a. It shows that the adsorption rate is higher at concentrations of 10 mg/L, 30 mg/L, and 50 mg/L, as the curve reaches equilibrium very quickly. In the case of 100 mg/L, the adsorption rate is slower, but the amount of dye adsorbed is greater due to the difference in dye concentrations. Figure 14b–d represent the graphs for the pseudo-first order, pseudo-second order, and intraparticle diffusion models, respectively. The gray shading in the graphs in Figure 14 indicates that the data from 0 to 30 min correspond to the equilibrium phase in the dark, during which degradation also occurred. The selection of the model that best describes the adsorption of NBB on the nanoparticles is based on the determination coefficient (R^2^) and the similarity between the experimental qe and the calculated qe, as shown in Table 1.

The adsorption kinetics of NBB onto KGM–CeO_2_ NPs were evaluated using pseudo-first-order (PFO), pseudo-second-order (PSO), and intra-particle diffusion (IPD) models to determine the adsorption mechanism and identify the model that best describes the adsorption process over a concentration range of 10–100 mg/L. From Table 1, it can be observed that the experimental equilibrium adsorption capacities (qe, exp) increased with increasing initial dye concentration, ranging from 6.79 mg g^−1^ at 10 mg/L to 60.44 mg g^−1^ at 100 mg/L, indicating concentration-dependent adsorption driven by increased mass transfer and stronger concentration gradients. Among the three kinetic models, the pseudo-second-order (PSO) model demonstrated the best overall fit for all tested concentrations, with exceptionally high correlation coefficients (R^2^ = 0.9929–1.0000). The PSO model also showed the closest agreement between calculated and experimental qe values, particularly at lower concentrations. For example, at 10 mg/L, qe, cal (6.82 mg g^−1^) closely matched qe, exp (6.79 mg g^−1^), while R^2^ reached 1.0000, indicating nearly perfect fitting. Even at higher concentrations, although qe, cal values were somewhat overestimated (e.g., 72.80 mg g^−1^ at 100 mg/L) compared with the experimental value (qe, exp = 60.44 mg g^−1^ at 100 mg/L), the very high R^2^ values strongly support PSO as the dominant kinetic model. These findings suggest that chemisorption is the primary adsorption mechanism, involving electron sharing or exchange between NBB molecules and the active functional groups on the surface of the KGM–CeO_2_ nanoparticles.

In contrast, the pseudo-first-order (PFO) model showed significantly poorer fitting, especially at lower concentrations, with R^2^ values ranging from 0.4743 to 0.9804. The calculated qe values were substantially lower than experimental values, particularly at 10 mg/L, where qe, cal was only 0.078 mg g^−1^ compared to qe, exp of 6.79 mg g^−1^. Additionally, the negative k1 values indicate poor applicability of the PFO model and suggest that first-order physical adsorption does not accurately represent the adsorption system. This confirms that simple physisorption is insufficient to explain NBB adsorption onto KGM–CeO_2_.

The intra-particle diffusion (IPD) model showed variable fit quality across concentrations. At lower concentrations (10 and 30 mg/L), the model produced relatively poor R^2^ values (0.6266 and 0.4636), suggesting minimal diffusion control. However, at 100 mg/L, the R^2^ value improved substantially (0.9777), indicating that intra-particle diffusion becomes increasingly relevant at higher dye concentrations. The increasing ki values from 0.00447 to 4.36197 mg g^−1^ min^−1/2^ with concentration further support enhanced diffusion as dye loading increases. Nevertheless, the large intercept values (C ≠ 0) at all concentrations indicate that boundary layer diffusion and surface adsorption also significantly contribute to the adsorption process. Since the IPD lines do not pass through the origin, intra-particle diffusion cannot be considered the sole rate-controlling mechanism.

In comparison to literature studies, very few reports are available on the degradation of Naphthol Blue Black using CeO_2_-based materials. One previous study reported that a Ce–Zn coupled oxide nanoparticle exhibited better photocatalytic degradation of NBB compared to individual CeO_2_ and ZnO NPs [58]. In contrast, the present study demonstrates that eco-friendly KGM–CeO_2_ nanoparticles show effective adsorption and photocatalytic degradation of NBB upto 99%, highlighting their potential for textile wastewater treatment.

#### 3.6.2. Adsorption Kinetics of MO

From Table 2, it can be observed that, among all tested models, the pseudo-second-order (PSO) model exhibited the highest correlation coefficients (R^2^ = 0.997–0.999) across all initial dye concentrations (10–100 mg/L), demonstrating a significantly better fit than both the pseudo-first-order and intra-particle diffusion models. Additionally, the calculated equilibrium adsorption capacities (qe, cal) from the PSO model closely matched the experimental qe values, confirming the reliability of this model in describing the adsorption system. For example, at 100 mg/L, the PSO-calculated qe (60.38 mg g^−1^) was highly consistent with experimental q_e_ (58.77 mg g^−1^) adsorption capacity, indicating strong predictive capability.

In contrast, the pseudo-first-order model showed comparatively lower R^2^ values (0.845–0.982), particularly at higher concentrations, and greater deviation between calculated and experimental qe values. This suggests that MO adsorption onto KGM–CeO_2_ cannot be adequately explained solely by physical adsorption or diffusion through a simple first-order process. The lower predictive accuracy indicates that external adsorption alone is insufficient to describe the adsorption pathway.

The intra-particle diffusion (IPD) model showed moderate to high R^2^ values (0.874–0.968), indicating that pore diffusion contributes to the adsorption process but is not the exclusive rate-limiting mechanism. The presence of significant intercept values (C ≠ 0) confirms that the adsorption process involves multiple steps, including boundary layer diffusion, surface adsorption, and subsequent diffusion into internal pores. Since the IPD plots did not pass through the origin, intra-particle diffusion cannot be considered the sole controlling step.

Table 3 presents a comparison of the photocatalytic performance of the synthesized KGM-CeO_2_ nanoparticles with previously reported CeO_2_-based photocatalysts for the degradation of methyl orange under various irradiation conditions. The synthesized KGM–CeO_2_ nanoparticles demonstrated high photocatalytic degradation efficiency (91%) using only 50 mg of adsorbent, showing competitive performance compared with many previously reported CeO_2_-based systems that often require metal doping or additional polymer modifications. The enhanced activity can be attributed to the biopolymer-assisted porous morphology, increased surface-active sites, improved dye adsorption capacity, and effective interaction between KGM and CeO_2_ nanoparticles.

#### 3.6.3. Adsorption Kinetics of Mixed Dye (MO-NBB)

The adsorption kinetics of the mixed dye(NBB-MO) system onto KGM–CeO_2_ NPs were analyzed using pseudo-first-order (PFO), pseudo-second-order (PSO), and intra-particle diffusion (IPD) models at an initial concentration of 100 mg/L to determine the adsorption mechanism under competitive multi-dye conditions. As shown in Table 4, the experimentally determined equilibrium adsorption capacity was qe, exp = 59.35 mg g^−1^, indicating that the KGM–CeO_2_ nanoparticles exhibit strong adsorption performance even in a mixed-dye system. This suggests that the material provides abundant active sites and favorable surface functionalities that can simultaneously interact with both dye molecules, despite possible competitive adsorption effects. Among all tested kinetic models, the pseudo-second-order (PSO) model provided the best fit for the mixed dye adsorption system. It showed an excellent correlation coefficient (R^2^ = 0.9997) and a calculated equilibrium adsorption capacity (qe, cal = 59.62 mg g^−1^), which closely matches the experimental value. This strong agreement confirms that the adsorption process is primarily governed by chemisorption involving valence forces, likely including electrostatic interactions, hydrogen bonding, and surface complexation between dye molecules and the functional groups (–OH, –NH) as well as CeO_2_ active sites of the nanoparticles. In contrast, the pseudo-first-order (PFO) model exhibited a lower goodness of fit (R^2^ = 0.963) and underestimated the adsorption capacity (qe, cal = 54.88 mg g^−1^). This deviation indicates that physisorption and external mass transfer are not sufficient to fully describe the adsorption kinetics, particularly in a system involving competitive adsorption between MO and NBB molecules.).

The intra-particle diffusion (IPD) model showed a relatively good correlation (R^2^ = 0.958), with a high diffusion rate constant (k_i_ = 2.864 mg g^−1^ min^−1/2^) and a significant intercept (C = 48.11). The non-zero intercept confirms that the IPD plots do not pass through the origin, indicating that intra-particle diffusion is involved in the adsorption process but is not the sole rate-limiting step. This suggests that the adsorption mechanism proceeds through multiple stages, including rapid external surface adsorption, boundary layer diffusion, and gradual diffusion into the internal porous structure of the adsorbent.

### 3.7. Comparative Adsorption Kinetics of NBB, MO, and Mixed NBB–MO Systems

A comparative evaluation of the adsorption kinetics of NBB, MO, and mixed NBB–MO dye systems onto KGM–CeO_2_ NPs, as shown in Table 5, revealed notable differences in adsorption affinity and mechanism. Among the three systems, NBB exhibited the highest overall adsorption affinity toward KGM–CeO_2_, followed by MO, while the mixed dye system showed slightly reduced adsorption performance due to competitive adsorption for active sites. The consistently high R^2^ values confirm that the overall adsorption mechanism remains governed by pseudo-second-order chemisorption kinetics. The superior affinity of NBB is likely attributed to its larger aromatic structure, multiple sulfonate groups, and greater availability of functional groups capable of stronger electrostatic attraction, hydrogen bonding, and surface complexation with the hydroxyl, amino, and CeO_2_ active sites of the nanoparticles. In contrast, MO showed slightly lower adsorption affinity, likely due to its comparatively smaller molecular structure and fewer interactive sites.

For all three dye systems, the pseudo-second-order (PSO) kinetic model consistently provided the best fit, with the highest correlation coefficients (R^2^ values approaching unity) and the closest agreement between calculated and experimental adsorption capacities. This confirms that chemisorption is the dominant adsorption mechanism for NBB, MO, and mixed NBB–MO adsorption, involving electron sharing or exchange between dye molecules and the multifunctional active surface of KGM–CeO_2_. The pseudo-first-order model showed comparatively weaker fitting, indicating that physisorption alone could not adequately explain the adsorption process. The intra-particle diffusion model suggested that diffusion within pores contributes to adsorption, particularly at higher concentrations, but was not the sole rate-determining step in any system. The presence of significant intercept values indicated that surface adsorption and boundary layer diffusion also play important roles. Overall, these findings demonstrate that KGM–CeO_2_ NPs possess strong multifunctional adsorption capability for both single and mixed dye systems, with particularly high affinity for NBB. The consistent suitability of the pseudo-second-order model across all systems highlights the importance of chemisorption-driven interactions, confirming the potential of KGM–CeO_2_ as an effective adsorbent for complex dye-contaminated wastewater treatment.

## 4. Conclusions

In the present study, KGM-capped CeO_2_ nanoparticles were successfully synthesized under different pH and temperature conditions to determine the optimal parameters for nanoparticle formation and photocatalytic performance. Comprehensive characterization using FTIR, XRD, SEM, EDX, TEM, UV–Vis spectroscopy, DLS, and zeta potential analysis confirmed the successful formation of stable KGM–CeO_2_ nanoparticles with distinct physicochemical properties influenced by synthesis conditions. Among the investigated conditions, the sample synthesized at pH 9 and 30 °C exhibited favorable optical and structural characteristics, with a band gap energy of 3.27 eV, indicating enhanced light absorption capability. XRD analysis confirmed the crystalline nature of the nanoparticles with crystallite sizes ranging from approximately 7–11 nm. SEM and TEM observations revealed a porous sponge-like morphology with branched chain-like irregular nanostructures, providing a high surface area favorable for adsorption and photocatalytic applications. DLS analysis indicated a polydisperse nanoparticle distribution with an average hydrodynamic size of approximately 28.8 nm, while zeta potential measurements confirmed excellent colloidal stability due to effective capping and stabilization by the KGM biopolymer, which minimized nanoparticle agglomeration.

The synthesized KGM–CeO_2_ nanoparticles demonstrated excellent adsorption and photocatalytic performance toward the degradation of textile dyes, including Naphthol Blue Black, Methyl Orange, and their mixed dye system. Adsorption studies showed removal efficiencies of up to approximately 60%, while photocatalytic degradation under UV irradiation for 120 min under acidic conditions with a dye concentration of 100 mg/L achieved degradation efficiencies of about 99% for NBB and 91% for MO. In contrast, the mixed NBB–MO system exhibited a comparatively lower degradation efficiency of approximately 52%, mainly due to competitive adsorption and active-site occupation between dye molecules. Adsorption equilibrium and kinetic studies revealed that the pseudo-second-order (PSO) model provided the best fit for all dye systems, indicating that chemisorption is the dominant adsorption mechanism. The kinetic behavior suggests that effective dye removal occurs through a synergistic combination of adsorption and photocatalytic irradiation processes. Furthermore, intra-particle diffusion analysis confirmed that adsorption proceeds through multiple stages involving surface adsorption, boundary layer diffusion, and pore diffusion mechanisms.

Collectively, these findings demonstrate that KGM–CeO_2_ nanoparticles synthesized through an eco-friendly and cost-effective approach possess excellent structural stability, adsorption capability, and photocatalytic efficiency for the removal of azo dye pollutants from wastewater. The consistent applicability of the pseudo-second-order kinetic model highlights the significance of chemisorption-driven interactions in the degradation process, confirming the strong potential of KGM–CeO_2_ nanoparticles as sustainable multifunctional materials for wastewater treatment and environmental remediation applications.

## Figures and Tables

**Table 1:** Kinetic parameters obtained from pseudo-first-order, pseudo-second-order, and intra-particle diffusion models for the adsorption of Naphthol Blue Black onto KGM–CeO_2_ nanoparticles.

Model	Parameters	Dye Concentrations (mg/L)			
		10 mg/L	30 mg/L	50 mg/L	100 mg/L
	q_e_ experimental (mg g^−1^)	6.7877	18.8030	30.7826	60.4417
**Pseudo-First Order**	k_1_ (min^−1^)	−0.00587	−0.03759	−0.04371	−0.04201
	q_e_ calculado (mg g^−1^)	0.07770	9.0533	22.6721	17.5690
	R^2^	0.47433	0.8695	0.98037	0.86997
**Pseudo-Second Order**	k_2_ (g mg^−1^ min^−1^)	0.14724	0.05275	0.03155	0.01435
	q_e_ calculado (mg g^−1^)	6.8179	21.1787	35.0389	72.8015
	R^2^	1	0.99995	0.99979	0.9929
**Intraparticle Diffusion**	k_i_ (mg g^−1^ min^−1/2^)	0.00447	0.2768	0.95561	4.36197
	C	6.74142	16.09406	21.0638	11.70471
	R^2^	0.62664	0.46355	0.78216	0.97773

**Table 2:** Kinetic parameters obtained from adsorption kinetic models for the adsorption of Methyl Orange onto KGM–CeO_2_ nanoparticles.

Model	Parameters	Dye Concentrations (mg/L)			
		10 mg/L	30 mg/L	50 mg/L	100 mg/L
**Pseudo First order (PFO)**	k1 (min^−1^)	0.018	0.071	0.112	0.032
	qe calculado (mg g^−1^)	5.95	21.09	45.53	61.64
	R^2^	0.901	0.956	0.982	0.845
**Pseudo-second order (PSO)**	k2 (g mg^−1^ min^−1^)	0.031	0.0087	0.0046	0.0019
	qe calculado (mg g^−1^)	6.02	18.11	30.42	60.38
	R^2^	0.999	0.999	0.998	0.997
**Intraparticle Diffusion (IPD)**	ki (mg g^−1^ min^−1/2^)	0.028	0.126	0.219	0.463
	C	5.79	17.18	28.41	57.42
	R^2^	0.874	0.913	0.941	0.968

**Table 3:** Comparison of previously reported literature studies on the photocatalytic degradation of Methyl Orange using CeO_2_-based nanomaterials and the present KGM–CeO_2_ nanoparticles system.

Photocatalyst Material	Morphology/Nature	Light Source	Degradation Efficiency (%)	Time (min)	Key Features	Reference
CeO_2_ nanoparticles	Nanoparticles	UV	87.5%	16	Oxygen vacancies, high stability	Soney et al., 2024 [59]
Magnetic biochar-CeO_2_	Porous magnetic nanoparticles	UV	96.7%	16	Biochar-assisted charge transfer	Soney et al., 2024 [60]
Ag/Sn-doped CeO_2_	Ag decorated nanoparticles	Visible	99%	45	LSPR effect, reduced band gap	Kazazi et al., 2019 [61]
CeO_2_ nanoparticles	Nanocrystalline particles	Sunlight	75%	80	pH-controlled synthesis	Gilani et al., 2024 [62]
CuCr_2_O_4_/CeO_2_	Heterojunction nanoparticles	LED light	~100%	30	Fenton-like catalytic pathway	Ghorai et al., 2021 [63]
CeO_2_ particles	Submicron particles	UV	~90%	—	Singlet oxygen generation	Minitha et al., 2015 [64]
Green synthesized CeO_2_	Spherical nanoparticles	Sunlight	98%	—	Plant extract mediated synthesis	Muthuvel et al., 2020 [65]
PS-CHO@CeO_2_	Core–shell microspheres	UV	93.03%	120	Improved Ce^3+^ concentration	Ni et al., 2021 [66]
GO/CeO_2_ nanoparticles	Spherical nanoparticles	Visible	93%	—	Enhanced charge separation	Iqbal et al., 2023 [67]
CeO_2_ nanocubes	Nanocube morphology	Visible	95%	90	Ce^3+^/Ce^4+^ redox enhancement	Latha et al., 2018 [68]
Cu/Bi co-doped CeO_2_	Hybrid nanostructure	Solar	95.79%	50	Improved conductivity and defects	Ishfaq et al., 2023 [22]
Biosynthesized CeO_2_	Nanoparticles	UV	93%	120	Reusable up to 6 cycles	Druzian et al., 2023 [69]
GO-CeO_2_ nanoparticles	Hydrothermal nanoparticles	Sunlight	94%	—	High TOC mineralization	Rauf et al., 2025 [70]
Fe-doped CeO_2_	Doped nanoparticles	Visible	88%	60	Reduced band gap	Vidhya et al., 2024 [71]
Nd-CeO_2_ nanoparticles	Defect-rich non-spherical particles	UV	95%	60	Efficient charge separation	Tamilveerapandiyan et al., 2026 [72]
ZnAl_2_O_4_-CeO_2_ (70:30)	Heterostructure composite	UV	98%	—	Reduced recombination	Dhinagaran et al., 2021 [73]
KGM-CeO_2_ nanoparticles	Biopolymer-capped porous aggregated nanoparticles	UV	91%	120	Eco-friendly synthesis, enhanced adsorption, and biopolymer stabilization	Present work

**Table 4:** Photocatalytic degradation performance of the mixed dye system containing Naphthol Blue Black and Methyl Orange using KGM–CeO_2_ nanoparticles under UV irradiation.

Model	Parameters	Dye Concentaration (mg/L)
		100 mg/L
	qe experimental (mg g^−1^)	59.3490
**Pseudo-First order**	k_1_ (min^−1^)	0.0079
	qe calculado (mg g^−1^)	54.88
	R^2^	0.963
**Pseudo-second order**	k_2_ (g mg^−1^ min^−1^)	0.0098
	qe calculado (mg g^−1^)	59.62
	R^2^	0.9997
**Intraparticle Diffusion**	kᵢ (mg g^−1^ min^−1/2^)	2.864
	C	48.11
	R^2^	0.958

**Table 5:** Comparative analysis of adsorption kinetic mechanisms for Naphthol Blue Black, Methyl Orange, and mixed dye systems onto KGM–CeO_2_ nanoparticles.

Dye Systems	Best Model	Evidence	Dominant Mechanism
**NBB**	Pseudo-second-order (PSO)	Highest R^2^ (0.9929–1.0000)	Chemisorption
**MO**	Pseudo-second-order (PSO)	Highest R^2^ (0.997–0.999)	Chemisorption
**NBB–MO**	Pseudo-second-order (PSO)	Highest R^2^ (0.9997)	Competitive chemisorption

## Data Availability

The original contributions presented in this study are included in the article/Supplementary Materials. Further inquiries can be directed to the corresponding authors.

## Supplementary Materials

The following supporting information can be downloaded at: https://www.mdpi.com/article/10.3390/nano16120739/s1, Figure S1: Nicomp particle size distribution analysis of KGM–CeO_2_ nanoparticles synthesized at pH 9 and 30 °C, showing volume-weighted and number-weighted distributions; Figure S2: Zeta potential analysis of KGM–CeO_2_ nanoparticles synthesized at pH 9 and 30 °C.

## Abbreviations

The following abbreviations are used in this manuscript:

KGM	Konjac Glucomannan
CeO_2_	Cerium oxide
NBB	Naphthol Blue Black
MO	Methyl Orange
CR	Congo Red
NPs	Nanoparticles
NBB-MO	Mixed dye system of Naphthol Blue Black and Methyl Orange
UV	Ultraviolet radiation
FTIR	Fourier Transform Infrared Spectroscopy
XRD	X-ray Diffraction spectroscopy
FESEM-EDS	Field Emission Scanning Electron Microscopy with Energy-Dispersive X-ray Spectroscopy
TEM	Transmission Electron microscopy
DLS	Dynamic light scattering
ZP	Zeta Potential
PFO	Pseudo First order
PSO	Pseudo-second order
IPD	Intra-particle Diffusion
