# Supplementary material for: Green Synthesis and Characterization of Konjac Glucomannan-Capped Cerium Nanoparticles for Photocatalytic Degradation of Naphthol Blue Black and Methyl Orange Dyes in Wastewater
Source: Nanomaterials (Basel). 2026 Jun 13;16(12):739. doi: 10.3390/nano16120739 (PMC13306176; doi:10.3390/nano16120739)
Supplement: Supplementary file 1 [file nanomaterials-16-00739-s001.zip › nanomaterials-4348460-supplementary.pdf]

Supplementary Figures

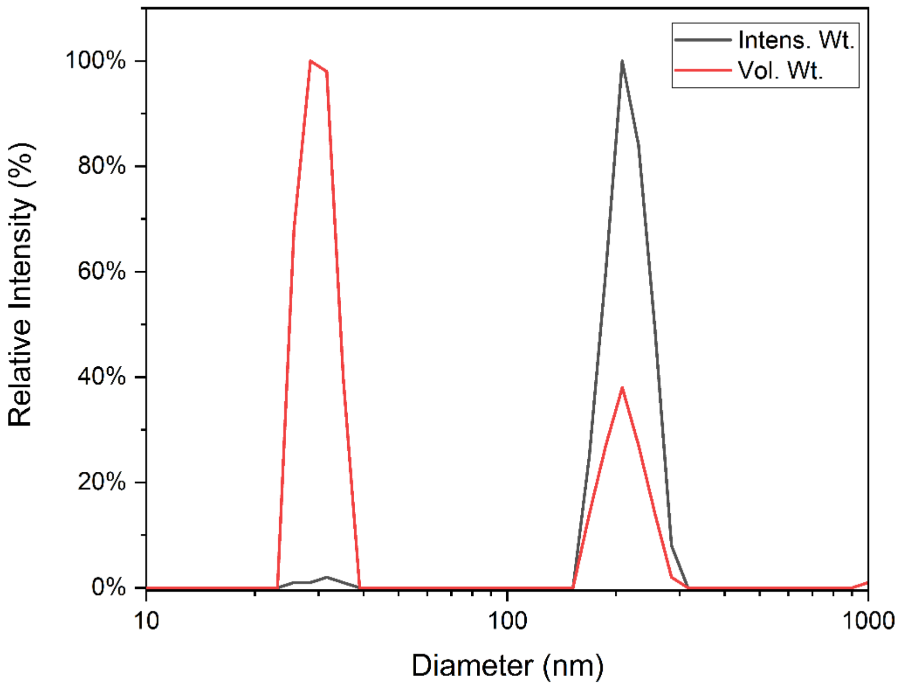

Figure S1. Nicomp particle size distribution analysis of KGM–CeO_2 nanoparticles synthesized at pH 9 and 30°C, showing volume-weighted and number-weighted distributions.

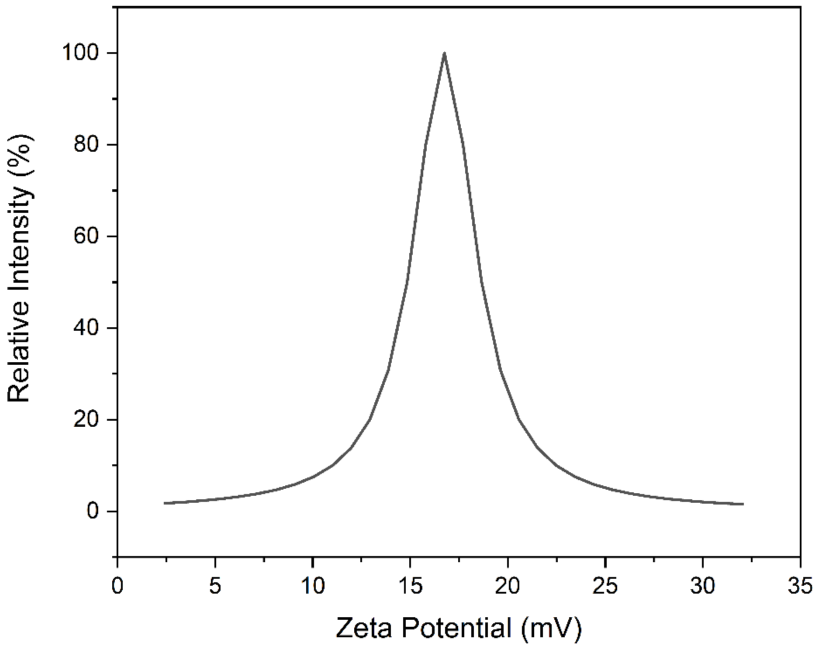

Figure S2. Zeta potential analysis of KGM–CeO_2 nanoparticles synthesized at pH 9 and 30°C
